# 

**Published:** 1872-11

**Authors:** 


### CONTENTS:

ORIGINAL COMMUNICATIONS:

ARTICLE I. General Review of Medical Progress and News. By J. H. Etheridge, M.D., Chicago ......... 641

ART. II. The Panaritium (Felon)—Consequences and Treatment. By Carl Proegler, M.D. (Continued)... 656

ART. III. History of the Linear Extraction of Cataract. By B. B. Schwarzbach, M.D., St. Paul ...... 660

ART. IV. Electrical Instruments for Medical Use. By P. S. Hayes, M. D., Chicago ..................... 665

EDITORS' BOOK TABLE................ 680

EDITORIAL:

Address of Prof. Allen, inaugurating Lecture Course of 1872-3, in Rush Med. College...................... 683

College Courant, New Haven........ 693

Chicago Medical Register and Directory for 1872-3..................... 694

LOOT......... ....................,..... 695

